# Fibrinogen is associated with glucose metabolism and cardiovascular outcomes in patients with coronary artery disease

**DOI:** 10.1186/s12933-020-01012-9

**Published:** 2020-03-19

**Authors:** Shuo-Lin Liu, Na-Qiong Wu, Hui-Wei Shi, Qian Dong, Qiu-ting Dong, Ying Gao, Yuan-Lin Guo, Jian-Jun Li

**Affiliations:** Endocrinology & Cardiometabolic Center, State Key Laboratory of Cardiovascular Disease, Fuwai Hospital, National Center for Cardiovascular Diseases, Chinese Academy of Medical Sciences and Peking Union Medical College, No 167 BeiLiShi Road, XiCheng District, Beijing, 100037 China

**Keywords:** Fibrinogen, HbA1c, Fasting blood glucose, Diabetes mellitus, Coronary artery disease, Prognosis

## Abstract

**Background:**

The present cohort study aims to examine the relationship between fibrinogen (Fib) levels and glucose metabolism [fasting blood glucose (FBG) and hemoglobin A1c (HbA1c)] and investigate the impact of high Fib on cardiovascular outcomes in patients with stable CAD and pre-diabetes mellitus (pre-DM) or diabetes mellitus (DM).

**Methods:**

This study included 5237 patients from March 2011 to December 2015. Patients were distributed into three groups according to Fib levels (low Fib, median Fib, high Fib) and further categorized by glucose metabolism status [normal glucose regulation (NGR), Pre-DM, DM]. All patients were followed up for the occurrences of major adverse cardiovascular events (MACEs), including cardiovascular mortality, nonfatal MI, stroke, and unplanned coronary revascularization.

**Results:**

Linear regression analyses showed that FBG and HbA1c levels were positively associated with Fib in overall CAD participants, either with or without DM (all *P *< 0.001). During an average of 18,820 patient-years of follow-up, 476 MACEs occurred. High Fib was independently associated with MACEs after adjusting for confounding factors [Hazard Ratio (HR): 1.57, 95% confidence interval (CI) 1.26–1.97, *P *< *0.001*]. Furthermore, DM but not pre-DM was a significant predictor of MACEs (*P *< 0.001 and *P *> 0.05, respectively). When patients were stratified by both glucose metabolism status and Fib levels, high Fib was associated with a higher risk of MACEs in pre-DM (HR 1.66, 95% CI 1.02–2.71, *P *< 0.05). Medium and high Fib levels were associated with an even higher risk of MACEs in DM (HR 1.86, 95% CI 1.14–3.05 and HR 2.28, 95% CI 1.42–3.66, all *P *< 0.05). After adding the combination of Fib and glucose status to the Cox model, the C-statistic was increased by 0.015 (0.001–0.026).

**Conclusions:**

The present study suggested that Fib levels were associated with FBG and HbA1c in stable CAD patients. Moreover, elevated Fib was independently associated with MACEs in CAD patients, especially among those with pre-DM and DM, suggesting that Fib may provide incremental value in the cardiovascular risk stratification of pre-DM and DM patients.

## Background

Fibrinogen (Fib), as a thromboplastic and inflammatory marker, facilitates blood viscosity, platelet aggregation, fibrin cross-linking, and plays a pivotal role in the progression of atherosclerosis [1, 2]. Previous studies showed that Fib was not only an indicator of subclinical cardiovascular diseases, such as coronary artery calcification and intima-media thickness of the carotid artery [3, 4], but was also independently associated with the development of coronary artery disease (CAD), hypertension, stroke, as well as adverse cardiovascular events [5–9].

The prevalence of type 2 diabetes mellitus (DM) and its cardiovascular complications has increased significantly worldwide [10]. In China, the prevalence of DM and prediabetes mellitus (pre-DM) were also steadily increasing, with a rate of DM in adults reaching 10.9% and pre-DM reaching 35.7% in 2013 [11]. Chronic, low-grade inflammation is an important predisposing factor for DM, and also contributes to the genesis of diabetes complications [12]. Fib, one of the subclinical inflammation biomarkers, was reported to increase before the onset of DM [13], and elevate from normal glucose regulation (NGR) over pre-DM to DM [14]. In the meanwhile, it has been demonstrated that Fib level was significantly associated with glucose metabolism [including fasting blood glucose (FBG) and glycosylated hemoglobinA1c (HbA1c), a measure of long-term glycemic control] in patients with acute coronary syndromes (ACS) [15]. Nevertheless, few reports have explored the relationship between serum Fib levels and glucose metabolism in patients with new-onset stable CAD. Moreover, Fib was also implicated in the presence of macrovascular complications and microvascular disorders in DM, [14, 16, 17] while there has been no study to investigate the impact of Fib levels on cardiovascular risk in individuals with impaired glucose regulation.

The present cohort study was conducted to examine the relationship between serum Fib levels and glucose metabolism, and investigate the combined effect of high Fib and pre-DM on cardiovascular outcomes in a large cohort of patients with stable CAD.

## Materials and methods

### Study design and population

**Fig. 1:**
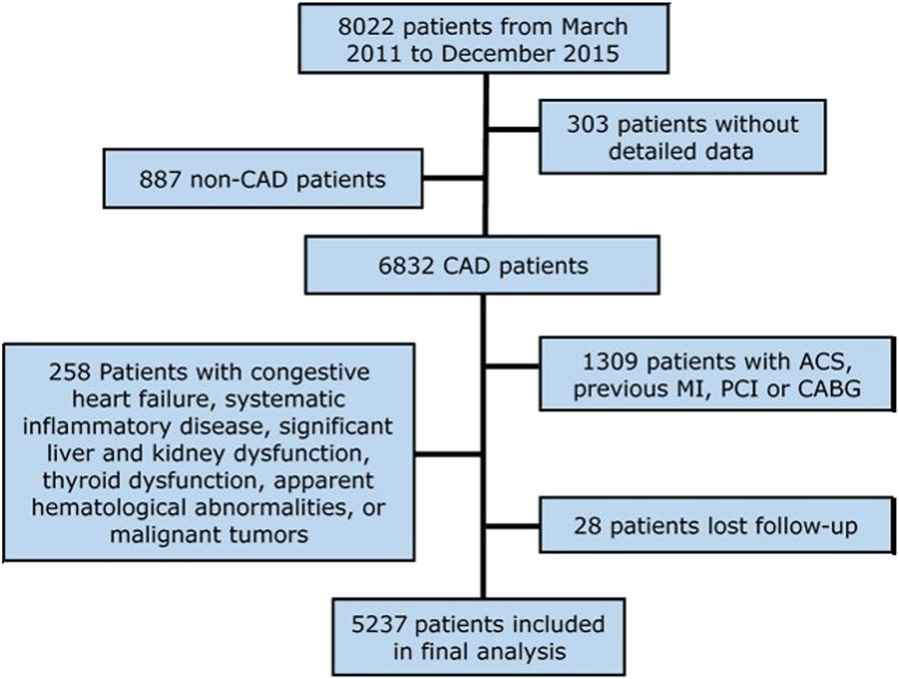
Flowchart of the study population. *ACS* acute coronary syndrome, *CAD* coronary artery disease, *PCI* percutaneous coronary intervention, *CABG* coronary artery bypass grafting

The present study was designed as a single-center, observational cohort study. As described in the flowchart (Fig. 1), from March 2011 to December 2015, 8022 patients who had received coronary angiography examination because of angina-like chest pain or positive noninvasive tests (such as treadmill exercise test or coronary computed tomography angiography) were recruited at the Fuwai Hospital, Chinese Academy of Medical Sciences. Then, 303 patients with the detailed data lost and 887 patients without angiographically determined CAD were excluded. Moreover, 1309 patients with acute coronary syndrome (ACS), previous myocardial infarction (MI), previous percutaneous coronary artery intervention (PCI) or coronary artery bypass grafting (CABG), 258 Patients with congestive heart failure, systematic inflammatory disease, significant liver and kidney dysfunction, thyroid dysfunction, apparent hematological abnormalities, or malignant tumors, and 28 patients lost to follow-up were excluded. Ultimately, 5237 patients were included in the final analysis. The patients were followed up regularly, as outlined in the study protocol. The study complied with the principles of the Declaration of Helsinki and was approved by the ethical review board of Fuwai Hospital (Beijing, China). Written informed consent was obtained from all participants.

All patients were followed up semiannually through telephone interviews or clinic visits. Trained clinical physicians or nurses who were blinded to previous medical histories accomplished the interview. All clinical events were carefully examined by three independent cardiologists. The major adverse cardiovascular events (MACEs) were cardiovascular mortality, nonfatal MI, stroke (hemorrhagic stroke or ischemic stroke), and unplanned coronary revascularization (PCI and CABG). Deaths of participants were informed by relatives, medical records, or physicians. The composite endpoints included cardiovascular mortality, nonfatal MI, and nonfatal stroke [18].

According to the American Diabetes Association criteria [19], DM was confirmed by a fasting blood glucose (FBG) level ≥ 7.0 mmol/L, or 2-h blood glucose level ≥ 11.1 mmol/L, or HbA1c level ≥ 6.5%, or currently using hypoglycemic medications. Pre-DM was defined as any nondiabetic patients who had an FBG ranges from 5.6 to < 7.0 mmol/L, or 2-h glucose ranges from 7.8 to < 11.1 mmol/L, or HbA1c level ranges from 5.7 to < 6.5%. NGR represented participants without pre-DM or DM.

### Laboratory tests

Blood samples were taken from patients in a fasting state for at least 12-h in the morning. The enzymatic hexokinase method was used to determine glucose concentrations. HbA1c was evaluated by Tosoh Automated Glycohemoglobin Analyser (HLC-723G8, Tokyo, Japan). The Fib levels were measured by a Stago auto-analyser with the STA Fibrinogen kit (Diagnostic Stago, 101 Taverny, France). All other laboratory parameters were analyzed at the biochemistry center of our hospital by standard biochemical tests.

### Statistical analysis

The statistical analyses were performed with SPSS version 22.0 software (SPSS Inc., Chicago, IL, USA) and R language version 3.5.2 (Eggshell Igloo). Missing values were dealt with multiple imputation method [20]. Continuous variables were presented as mean ± standard deviation (SD) or median (interquartile range). Categorical variables were presented as number (percentage). The distributions of parameters were examined by the Kolmogorov–Smirnov test. *P* values for trend across Fib levels in the continuous variables were tested by a generalized linear model. The post hoc multiple comparisons among groups were analyzed by Student’s *t*-test, one-way ANOVA or Mann–Whitney U test where appropriate. P values for trend in the categorical variables were compared by Chi-square test or Fisher exact test. Linear regression analyses were performed to measure the independent relationships between HbA1c and Fib or FBG and Fib. The event-free survival rates among groups were determined by the Kaplan–Meier method and compared by the log-rank test. Univariate and multivariate Cox proportional hazard regressions were used to calculate the hazard ratio (HR) and 95% confidence interval (CI). The adjusted Cox models were adjusted for risk factors as follows: age, sex, body mass index (BMI), smoking, hypertension, family history of coronary artery disease, left ventricular ejection fraction (LVEF), low density lipoprotein cholesterol (LDL-C), high lipoprotein cholesterol (HDL-C), Ln-transformed triglyceride (TG), Ln-transformed high-sensitivity C-reactive protein (HsCRP), and creatinine. The proportional hazard assumption for each Cox regression models was tested using Schoenfeld residuals and checked with smoothed plots of Schoenfeld residuals (all variables satisfy the PH assumption of Cox models). C-statistic and ΔC-statistic were calculated to evaluate the efficiency of models and the incremental value of adding the combination of Fib and glucose metabolism status into the original model. A value of *P *< 0.05 was considered statistically significant.

## Results

### Baseline characteristics

Patients were distributed into three groups, according to Fib levels. The baseline characteristics of the study population are shown in Table 1. Patients with higher Fib levels were more likely to be the female and the elderly, had higher BMI, FBG and HbA1c levels, higher lipid levels (including TC, LDL-C, and TG) and HsCRP levels, but lower LVEF (all *P *< 0.05) than those with low Fib levels. Moreover, the percentages of hypertension and DM were higher (all *P *< 0.001) in patients with higher Fib levels, while the percentages of current smokers and drinkers were lower (all *P *= 0.002) with the increase of Fib level. There were also significant differences in medications at discharge among the three groups. However, no significant differences were observed regarding the family history of CAD, HDL-C, and creatinine among the participants (all *p *> 0.05).

**Table 1:** Baseline characteristics of study population according to fibrinogen levels

	Total (n = 5237)	Low Fib (< 2.82 mg/dL) (n = 1746)	Medium Fib (2.82–3.39 mg/dL) (n = 1746)	High Fib (> 3.39 mg/dL) (n = 1745)	p-value for trend
Age, years	57.79 ± 10.11	56.23 ± 9.91	57.86 ± 9.97	59.28 ± 10.21	< 0.001
Male, n (%)	3729 (71.2)	1372 (78.6)	1241 (71.1)	1113 (63.8)	< 0.001
BMI (kg/m^2^)	25.78 ± 3.17	25.65 ± 3.08	25.76 ± 3.02	25.92 ± 3.39	0.041
Hypertension, n (%)	3320 (63.4)	1047 (60.0)	1114 (63.8)	1158 (66.4)	< 0.001
Diabetes, n (%)	1637 (31.3)	445 (25.5)	567 (32.5)	625 (35.8)	< 0.001
Family history of CAD, n (%)	812 (15.5)	276 (15.8)	267 (15.3)	267 (15.3)	0.916
Current Smokers, n (%)	2807 (53.6)	981 (56.2)	946 (54.2)	879 (50.4)	0.002
Drinkers, n (%)	1628 (31.1)	597 (34.2)	529 (30.3)	502 (28.8)	0.002
FBG (mmol/L)	5.75 ± 1.72	5.52 ± 1.53	5.74 ± 1.68	5.98 ± 1.90	< 0.001
HbA1c (%)	6.33 ± 1.10	6.09 ± 0.92	6.35 ± 1.09	6.54 ± 1.23	< 0.001
Creatinine (μmol)	76.61 ± 18.68	15.92 ± 0.38	17.64 ± 0.42	21.96 ± 0.53	0.297
TC (mmol/L)	4.17 ± 1.16	4.00 ± 1.07	4.19 ± 1.14	4.31 ± 1.24	< 0.001
HDL-C (mmol/L)	1.05 ± 0.28	1.05 ± 0.28	1.04 ± 0.27	1.06 ± 0.30	0.463
LDL-C (mmol/L)	2.54 ± 1.00	2.41 ± 0.95	2.54 ± 0.95	2.66 ± 1.07	< 0.001
TG (mmol/L)	1.52 (1.13–2.12)	1.48 (1.06–2.04)	1.55 (1.14–2.20)	1.53 (1.17–2.87)	0.001
HsCRP, mg/dL	1.46 (0.73–2.98)	0.83 (0.49–1.39)	1.38 (0.75–2.43)	3.20 (1.65–7.58)	< 0.001
LVEF (%)	64.56 ± 7.29	65.13 ± 6.94	64.86 ± 6.87	63.68 ± 7.95	< 0.001
Medications at discharge					
Statins, n (%)	5106 (97.5)	1707 (97.8)	1699 (97.3)	1699 (97.4)	0.538
Aspirin, n (%)	5163 (98.6)	1716 (98.3)	1721 (98.6)	1724 (98.8)	0.524
β-blockers, %	4137 (79.0)	1365 (78.2)	1347 (77.2)	1422 (81.5)	0.004
ACEI/ARB, %	2602 (49.7)	813 (46.6)	860 (49.3)	928 (53.2)	< 0.001
CCB, n (%)	2115 (40.4)	689 (39.5)	738 (42.3)	685 (39.3)	0.134

The data are presented as mean ± standard deviation, median (interquartile range) or number (%). *BMI* body mass index, *CAD* coronary artery disease, *HbA1c* glycosylated hemoglobin, *FBG* fasting blood glucose, *TC* total cholesterol *HDL-C* high-density lipoprotein cholesterol, *LDL-C* low density lipoprotein cholesterol, *TG* triglyceride, *HsCRP* high-sensitivity C-reactive protein, *LVEF* left ventricular ejection fraction, *ACEI* angiotensin-converting enzyme inhibitors, *ARB* angiotensin receptor blockers, *CCB* calcium channel blockers

### Association of glucose metabolism with Fib

**Fig. 2:**
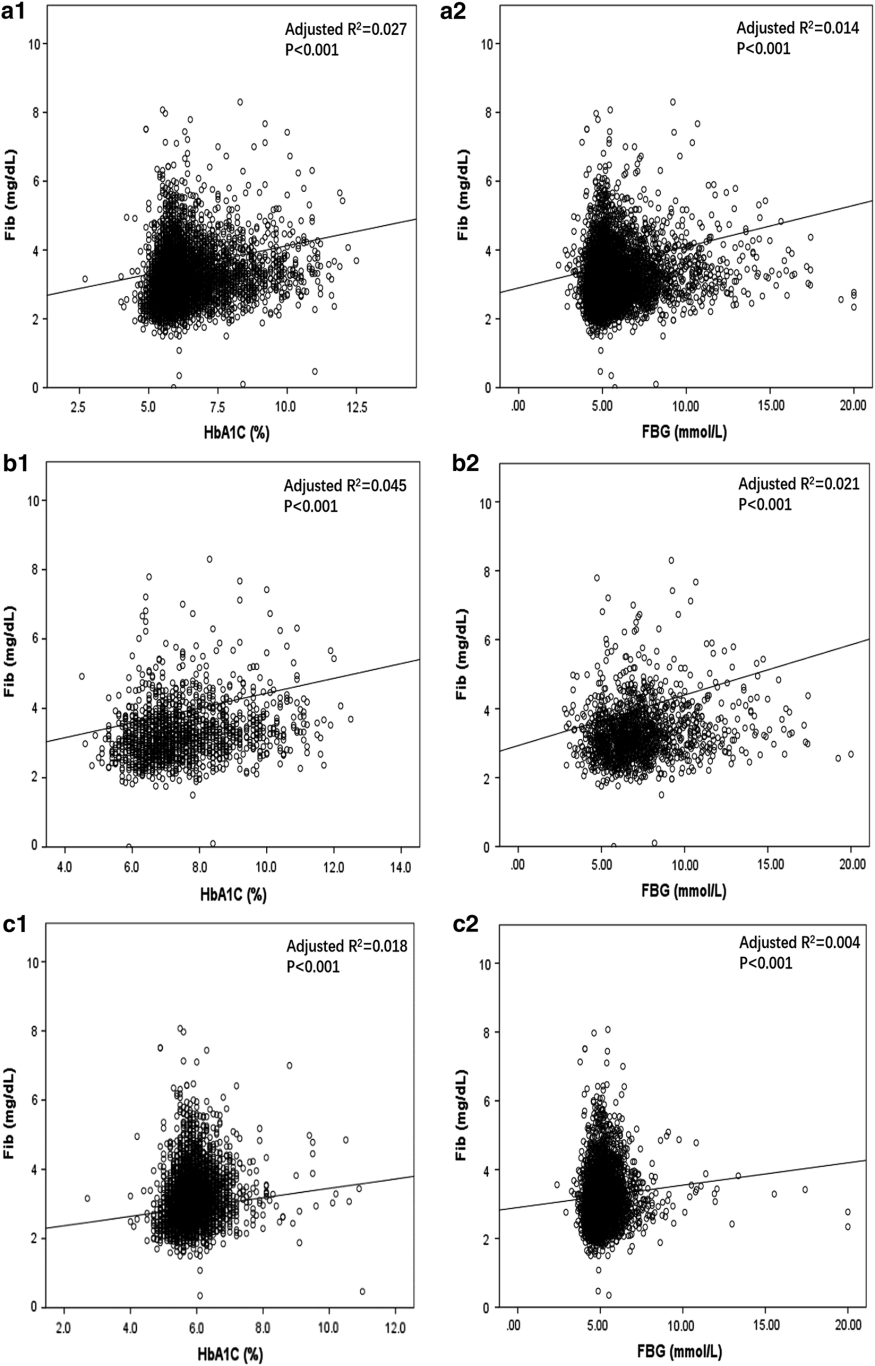
Linear regression analysis of the relationship between glucose metabolism and FIB. **a** Linear regression analysis of the relationship between glucose metabolism [HbA1c (**a1**), FBG (**a2**)] and FIB in overall participants with CAD. **b** Linear regression analysis of the relationship between glucose metabolism [HbA1c (**b1**), FBG (**b2**)] and FIB in CAD patients with DM. **c** Linear regression analysis of the relationship between glucose metabolism [HbA1c (**c1**), FBG (**c2**)] and FIB in CAD patients without DM. *FIB* fibrinogen, *DM* diabetes mellitus, *HbA1c* HaemoglobinA1c, *FBG* fasting blood glucose

Linear regression analyses were performed to explore the association between glucose metabolism indexes (FBG and HbA1c) and Fib (Table 2). HbA1c level (*R*^2^ = 0.027; Standard *β *= 0.166, *P *< 0.001) and FBG (*R*^2^ = 0.014; Standard *β *= 0.120, *P *< 0.001) were positively associated with Fib in overall participants with CAD. Furthermore, in CAD patients with DM, HbA1c level (*R*^2^ = 0.045; Standard *β *= 0.213, *P *< 0.001) and FBG level (*R*^2^ = 0.021; Standard *β *= 0.146, *P *< 0.001) were also significantly positively associated with Fib. Additionally, linear regression analysis indicated positive associations of Fib with HbA1c (*R*^2^ = 0.018; Standard *β *= 0.135, *P *< 0.001) and FBG (*R*^2^ = 0.004; Standard *β *= 0.065, *P *< 0.001) in non-DM population (Fig. 2).

**Table 2:** Linear regression analysis between glucose metabolism and fibrinogen in patients with stable coronary disease

Variable	Adjusted R^2^	Standard β	SEM	*P* value
Overall				
HbA1C, %	0.027	0.166	0.010	< 0.001
FBG, mmol/L	0.014	0.120	0.006	< 0.001
DM				
HbA1C, %	0.045	0.213	0.016	< 0.001
FBG, mmol/L	0.021	0.146	0.009	< 0.001
Non-DM				
HbA1C, %	0.018	0.135	0.022	< 0.001
FBG, mmol/L	0.004	0.065	0.013	< 0.001

*DM* diabetes mellitus, *HbA1c* Hemoglobin A1c, *FBG* fasting blood glucose, *CI* confidence interval, *SEM* standard error of estimate

### Fib levels and cardiovascular outcomes

Over an average of 18,820 patient-years of follow-up, 476 MACEs occurred (52 experienced cardiac death, 62 suffered nonfatal MI, 131 had strokes, and 231 received unplanned revascularization). The corresponding prevalence of MACEs in the low Fib, medium Fib, and high Fib group was 7.2%, 9.2%, and 10.9%, respectively. Univariate Cox proportional hazard regression analyses revealed that per SD change of Fib (HR: 1.18, 95% CI 1.09–1.27, *P *< 0.001), medium Fib (HR: 1.30, 95% CI 1.03–1.64, *P *= 0.029), high Fib (HR: 1.57, 95% CI 1.26–1.97, *P *< 0.001) were significantly associated with increased MACEs. After adjusting for potential confounders including age, sex, BMI, smoking, hypertension, family history of CAD, LVEF, LDL-C, HDL-C, Ln-transformed TG, Ln-transformed HsCRP, and creatinine, the multivariate cox proportional hazard regression analysis showed that Fib was also independently associated with MACEs (per SD change of Fib: HR: 1.11, 95% CI 1.01–1.24, *P *= 0.034, medium Fib: HR: 1.15, 95% CI 0.90–1.47, *P *= 0.274, high Fib: HR: 1.34, 95% CI 1.02–1.75, *P *= 0.035). Moreover, Fib was associated with composite endpoints including cardiovascular mortality, nonfatal MI, and nonfatal stroke (per SD change of Fib: HR: 1.17, 95% CI 1.02–1.34, *P *= 0.023, medium Fib: HR: 1.24, 95% CI 0.87–1.77, *P *= 0.243, high Fib: HR: 1.67, 95% CI 1.14–2.45, *P *= 0.008, Table 3) after adjustment for other variables.

**Table 3:** Relation of the fibrinogen level and cardiovascular outcomes in univariate and multivariate survival analysis

Variables	Univariate Cox regression		Multivariate Cox regression	
	Hazard ratio (95% CI)	P value	Hazard ratio (95% CI)	P value
MACE				
Fib (per SD change)	1.18 (1.09–1.27)	< 0.001	1.11 (1.01–1.24)	0.034
Low Fib	Reference	–	Reference	–
Medium Fib	1.30 (1.03–1.64)	0.029	1.15 (0.90–1.47)	0.274
High Fib	1.57 (1.26–1.97)	< 0.001	1.34 (1.02–1.75)	0.035
Composite endpoints				
Fib (per SD change)	1.26 (1.13–1.40)	< 0.001	1.17 (1.02–1.34)	0.023
Low Fib	Reference	–	Reference	–
Medium Fib	1.49 (1.06–2.10)	0.022	1.24 (0.87–1.77)	0.243
High Fib	2.05 (1.49–2.84)	< 0.001	1.67 (1.14–2.45)	0.008

*Fib* fibrinogen. Model adjusted for age, sex, body mass index, smoking, hypertension, family history of coronary artery disease, left ventricular ejection fraction, low density lipoprotein cholesterol, high lipoprotein cholesterol, Ln-transformed triglyceride, Ln-transformed high-sensitivity C-reactive protein, and creatinine

### Glucose metabolism, Fib levels, and cardiovascular outcomes

**Fig. 3:**
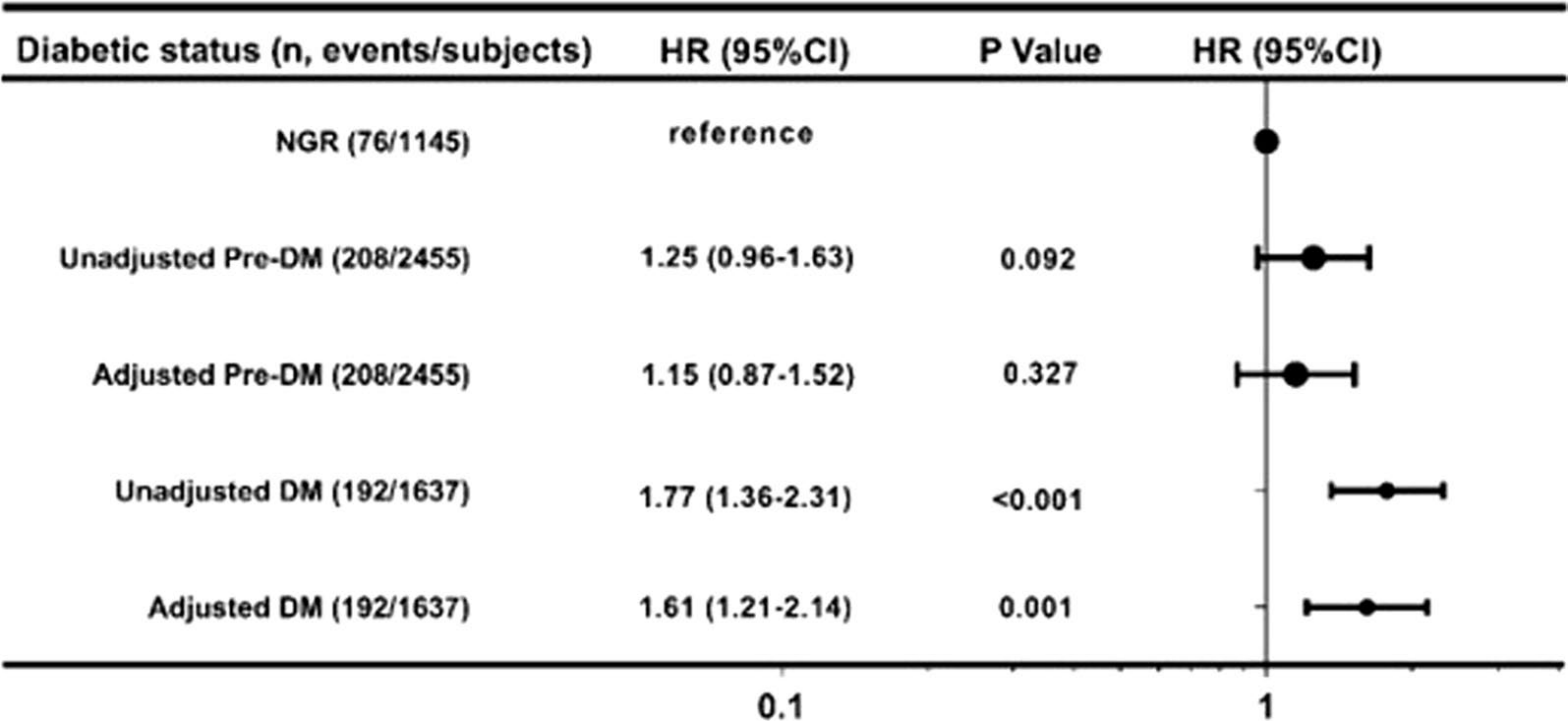
Relation of the different glucose metabolism status and cardiovascular outcomes in univariate and multivariate survival analysis. Univariate and multivariate Cox proportional hazards regression analysis was performed to test statistical significance. Model adjusted for age, sex, body mass index, smoking, hypertension, family history of coronary artery disease, left ventricular ejection fraction, low density lipoprotein cholesterol, high lipoprotein cholesterol, Ln-transformed triglyceride, Ln-transformed high-sensitivity C-reactive protein, and creatinine. *NGR* normal glucose regulation, *Pre-DM* pre-diabetes mellitus, *DM* diabetes mellitus

Over a median follow-up time of 3.3 years (2.8 to 5.1 years), the incidence rates of MACEs in Pre-DM (8.5%) and DM (11.7%) groups were higher than those in the NGR (6.6%) group (*p *< 0.001). Univariate Cox regression models showed that a baseline DM had a 1.77-fold higher risk of MACEs compared with the NGR group (HR: 1.77, 95% CI 1.36–2.31, *p *< 0.001). Additional adjustment for other variables only slightly attenuated this association. However, the presence of pre-DM did not elevate MACEs risk compared with the NGR group in any adjusted model (*P *> 0.05) (Fig. 3). Moreover, we further assessed the risk of MACEs stratified according to both glucose metabolism status and Fib levels (Table 4). High Fib did significantly elevate the risk of MACEs in pre-DM when compared with the reference group, with adjusted HR of 1.61 (95% CI 1.01–2.59, *P *< 0.05). The risks of MACEs in DM with medium Fib or high Fib groups were even higher, up to 1.86-fold (95% CI 1.14–3.05, *P *< 0.05) and 2.28-fold (95% CI 1.42–3.66, *P *< 0.05), compared with NGR and low Fib group.

**Table 4:** Fibrinogen levels in relation to cardiovascular events in patients with different glucose metabolism status

	Events/subjects	HR (95% CI)	
	476/5237	Crude model	Adjusted model
NGR			
Low Fib	25/510	Reference	Reference
Medium Fib	28/365	1.56 (0.91–2.67)	1.47 (0.85–2.52)
High Fib	23/270	1.81 (1.03–3.18)*	1.56 (0.87–2.80)
Pre-DM			
Low Fib	55/791	1.36 (0.85–2.19)	1.24 (0.78–2.00)
Medium Fib	72/814	1.81 (1.15–2.85)*	1.58 (0.99–2.51)
High Fib	81/850	1.95 (1.25–3.05)*	1.61 (1.01–2.59) *
DM			
Low Fib	45/445	2.09 (1.28–3.41)*	1.81 (1.13–2.92) *
Medium Fib	60/567	2.15 (1.35–3.43)*	1.86 (1.14–3.05) *
High Fib	87/625	2.89 (1.85–4.50)*	2.28 (1.42–3.66) *

Model adjusted for age, sex, body mass index, smoking, hypertension, family history of coronary artery disease, left ventricular ejection fraction, low density lipoprotein cholesterol, high lipoprotein cholesterol, Ln-transformed triglyceride, Ln-transformed high-sensitivity C-reactive protein, and creatinine. * p-value < 0.05. *Fib* fibrinogen, *NGR* normal glucose regulation, *Pre-DM* pre-diabetes mellitus, *DM* diabetes mellitus

**Fig. 4:**
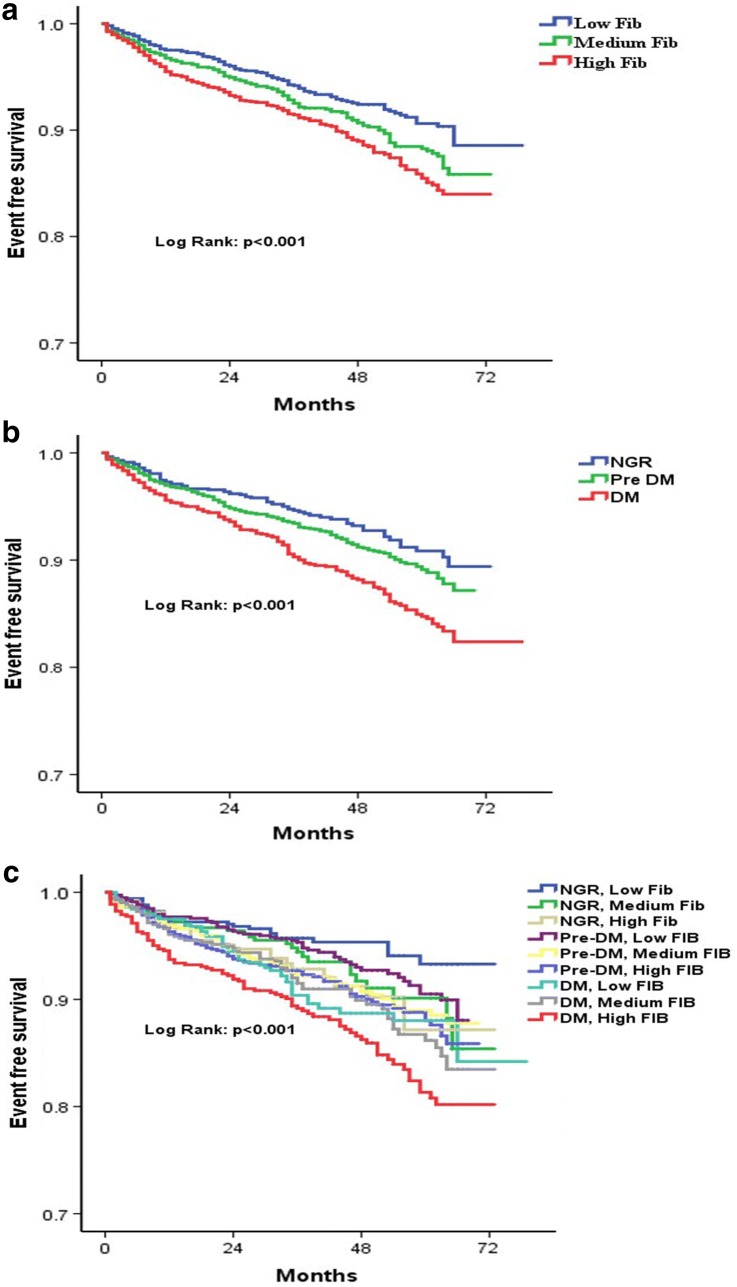
Kaplan-Meier analysis according to different fibrinogen levels (**a**), glucose metabolism status (**b**), and status of both fibrinogen levels and glucose metabolism (**c**)

Kaplan–Meier analysis with the Log-rank test indicated that patients with high Fib had the lowest cumulative event-free survival rates (Fig. 4a). Participants who had DM were least likely to be free of events among the three groups (*P *< 0.001 for all comparisons). Nevertheless, there was no difference in the rate of incident CVD outcomes between pre-DM and NGR groups (*P *= 0.091, Fig. 4b). Furthermore, we have assessed the prognostic significance of combining glucose metabolism and Fib levels, pre-DM with medium Fib, pre-DM with high Fib, DM with medium Fib, and DM with high Fib groups had lower cumulative incidence of MACE during follow-up compared with the reference group (NGR and low Fib group, all *P *< 0.01 respectively, Fig. 4c).

Finally, we evaluated the combined effect of Fib and glucose status on predicting the risk of MACE occurrence. C-statistic values increased from 0.598 (95% CI 0.571–0.625) for original prediction models of traditional risk factors to 0.613 (95% CI 0.586–0.640) for the combination of Fib and glucose status [ΔC-statistic: 0.015 (0.001–0.026), *P *= 0.022, Table 5].

**Table 5:** Incremental predictive values of glucose metabolism status and fibrinogen levels for cardiovascular outcomes

	C-statistic (95% CI)	∆C-statistic (95% CI)	p-value
Original model	0.598 (0.571–0.625)	–	–
Original model + GF	0.613 (0.586–0.640)	0.015 (0.001–0.026)	0.022

C-statistic and ∆C-statistic were used to interpret efficiency of the models and the incremental value of adding fibrinogen levels into original model. Original model included age, sex, body mass index, smoking, hypertension, family history of coronary artery disease, left ventricular ejection fraction, low density lipoprotein cholesterol, high lipoprotein cholesterol, Ln-transformed triglyceride, Ln-transformed high-sensitivity C-reactive protein, and creatinine. Original Model indicates the C-statistic for MACEs (cardiovascular mortality, non-fatal myocardial infarction, stroke and post-discharge unplanned revascularization)

## Discussion

In this cohort study, we found that serum Fib level was significantly associated with glucose metabolism (including FBG and HbA1c) in Chinese stable CAD patients undergoing coronary angiography with or without DM. Cox regression analysis revealed that Fib was independently associated with MACEs during a median follow-up of 3.3 years. Interestingly, DM but not pre-DM was a significant predictor of MACEs when stratified by glucose metabolism status. However, patients with pre-DM and high Fib (> 3.39 mg/dL) did have a 1.66-fold elevated risk of MACEs than those with normal blood glucose and low Fib. Moreover, adding the combination of Fib and glucose status to the model significantly improved the risk prediction for MACEs in the overall population. Our result, for the first time, provides evidence that patients with high Fib and pre-DM are prone to have a worse clinical prognosis.

### Fib and coronary artery disease

Fib is a central factor in the chronic inflammatory process of atherosclerosis. As an essential component in atherosclerotic plaques, Fib can cause migration, the proliferation of smooth muscle cells, and increasing levels of proinflammatory cytokines, including IL-6 and TNF-α [21]. It also alters the integrity of endothelial cell layer and vascular wall permeability through ICAM-1 and P-selection, lures leukocytes form blood to the vessel wall through ligand-receptor, induces accumulation and activation of platelets [22], therefore orchestrates a multicellular inflammatory cascade with chronically detrimental clinical manifestations to some extent [23]. Consequently, epidemiological studies have demonstrated that Fib was significantly associated with cardiovascular outcomes in patients with CAD. AtheroGene study enrolled 1806 stable CAD patients with a median follow-up time of 3.5 years and found that per SD change of Fib was associated with 1.27-fold increased risk of cardiovascular death and non-fatal myocardial infarction [24]. Similarly, Mahmud et al. [25] reported that Fib ≥ 280 mg/dl was associated with a 2.65-fold elevated risk of 1-year major cardiovascular events after PCI. Whereas, the EPIC-Norfolk study included 16,850 patients who had no known cardiovascular history and showed that serum Fib lost its significance as a predictor for the seventeen-year risk of cardiovascular mortality after adjusted for other inflammation markers [26]. Despite the conflicting results of Fib and cardiovascular outcomes, the Canakinumab Antiinflammatory Thrombosis Outcomes Study (CANTOS) [27] and Colchicine Cardiovascular Outcomes Trial (COLCOT) [28] did provide encouragement that antiinflammation treatment may be useful in coronary artery disease. Thus, more evidence for the value of Fib in cardiovascular risk stratification in different subgroups were needed.

### Fib, glucose metabolic disorder, and diabetic vascular complications

Concerning the relationship between Fib and glucose metabolic disorders, it has been demonstrated that Fib functioned as a crucial modulator of hemostatic balance and inflammatory processes in diabetes [22, 29]. Impaired glucose metabolism and the status of insulin resistance result in oxidative stress, inflammatory response, coagulation activation, and accelerated atherosclerosis pathogenesis [30]. Fib synthesis was significantly promoted in pre-DM and type 2 diabetes, [17, 31] especially in those with high levels of polyhedral erythrocytes [32]. HbA1c was reported to have a positive effect on Fib level in DM [33]. Zhang et al. [15] found that Fib was associated with FBG and HbA1c in patients with ACS and DM, but not in those without DM. However, it must be emphasized that the result should be appraised with caution due to the small sample size (103 DM and 308 non-DM). On the contrary, our results demonstrated that Fib was significantly associated with FBG and HbA1c in 5237, new-onset CAD patients either with or without DM (1637 DM and 3600 non-DM). Another reason for the conflicting result may be the difference in the study population, ACS versus stable CAD.

Furthermore, what is actually more important is whether Fib exerts an independent role in augmenting the risk of diabetic vascular complications. Fib independently associated with long-term microvascular disorders of type 2 DM, including diabetic nephropathy [34] and diabetic retinopathy (DR) [17], which increased the risk of cardiovascular events [35]. Interestingly, the Veterans Affairs Diabetes Trial [17] found that intensive glycemic control was associated with a decrease in the progression of DR in DM participants with lower Fib levels but not in DM patients with higher Fib levels, which suggests that there exists an interaction between glycemic control and Fib level.

### Fib and cardiovascular outcomes in patients with coronary artery disease and different glucose metabolism status

Glucose metabolism abnormalities are usual in patients with stable CAD and associated with a worse prognosis. Patients with CAD but without apparent glucose abnormalities were recommended to evaluate their glycemic state [36]. Prediabetes was associated with a worse prognosis in individuals with stable CAD only when combined with other cardiometabolic disorders [37–39]. Several previous studies demonstrated that the measurement of Fib provided incremental value to risk assessment in CAD patients with DM. The Gargano Heart Study has shown that Fib was an independent predictor of adverse major cardiovascular outcomes, including non-fatal myocardial infarction, stroke, and cardiovascular deaths, in 320 CAD patients with DM during an average follow-up time of 64.2 months [40]. That relationship was then validated in 1466, angiographic-proven stable CAD patients with DM over an average follow-up of 20.2 months [41] and 308 ACS patients with DM during a median follow-up of 27.5 months [15]. The Casale Monferrato Study demonstrated that Fib significantly associated with both 11-year all-cause and cardiovascular mortality in type 2 diabetes [42]. However, ADVANCE Study performed a nested case-cohort study in 2865 patients with DM and cardiovascular diseases and showed that IL-6 levels but not Fib levels added predictive value to diabetic macrovascular events and mortality [43]. The conflicting results between ADVANCE study and our study may due to its study population (including participants with stable CAD or MI). As the European Society of Cardiology emphasized recently, the dynamic nature of the CAD progression leads to different clinical presentations, which can have long, stable periods but can also become unstable at any time, typically attributed to an acute atherothrombotic event due to plaque rupture or erosion [44]. Thus, Fib levels may play various roles in ACS and chronic coronary syndromes (CCS). Besides, the predictive ability of Fib even changed over time in the DM population with ACS [15]. Nonetheless, there was no study available to evaluate the association between Fib levels and cardiovascular events in pre-DM status currently. To our best knowledge, this is the first study to investigate the combined effect of Fib and glucose metabolic status on MACEs risk. We found that patients with DM and medium Fib or high Fib had 1.86-fold and 2.28-fold increased risk of MACEs during the follow-up. Moreover, pre-DM alone was not an independent predictor of cardiovascular risk, but high Fib and pre-DM did have a worse cardiovascular prognosis, suggesting subclinical inflammation affects the prognosis of participants with impaired glucose metabolism.

The present study had several limitations. Firstly, since this is a single-center observational cohort study, some unmeasured confounders from the non-random assignment of exposure might influence the results of this study. Secondly, longer follow-up duration and larger sample population in this study will be required to investigate the prognostic value of pre-DM alone or combination with Fib in the long-term outcomes. Thirdly, this study only enrolled participants with stable CAD, which indicates that our results may only be applicable to stable CAD patients.

## Conclusions

Fib levels were associated with FBG and HbA1c and could be used as an independent predictor of MACEs in stable CAD patients. Patients with pre-DM and high Fib but not pre-DM alone had a worse prognosis. Furthermore, patients with DM and high Fib had the highest risk of MACEs. Fib may provide incremental value in the cardiovascular risk stratification of pre-DM and DM patients.

## Data Availability

The datasets used and/or analysed during the current study are available from the corresponding author on reasonable request.

## Abbreviations

CAD: Coronary artery disease
DM: Diabetes mellitus
Pre-DM: Prediabetes mellitus
NGR: Normal glucose regulation
FBG: Fasting blood glucose
HbA1c: Glycosylated hemoglobinA1c
ACS: Acute coronary syndromes
Fib: Fibrinogen
MI: Myocardial infarction
PCI: Percutaneous coronary artery intervention
CABG: Coronary artery bypass grafting
MACEs: Major adverse cardiovascular events
BMI: Body mass index
LVEF: Left ventricle ejection fraction
LDL-C: Low density lipoprotein cholesterol
HDL-C: High density lipoprotein cholesterol
TG: Triglyceride
HsCRP: High-sensitivity C-reactive protein
DR: Diabetic retinopathy
